# *H19* long non-coding RNA contributes to sphere formation and invasion through regulation of CD24 and integrin expression in pancreatic cancer cells

**DOI:** 10.18632/oncotarget.26176

**Published:** 2018-10-05

**Authors:** Norihiko Sasaki, Masashi Toyoda, Hisashi Yoshimura, Yoko Matsuda, Tomio Arai, Kaiyo Takubo, Junko Aida, Toshiyuki Ishiwata

**Affiliations:** ^1^ Research Team for Geriatric Medicine (Vascular Medicine), Tokyo Metropolitan Institute of Gerontology, Tokyo 173-0015, Japan; ^2^ Division of Physiological Pathology, Department of Applied Science, School of Veterinary Nursing and Technology, Nippon Veterinary and Life Science University, Tokyo 180-8602, Japan; ^3^ Department of Pathology, Tokyo Metropolitan Geriatric Hospital and Institute of Gerontology, Tokyo 173-0015, Japan; ^4^ Division of Aging and Carcinogenesis, Research Team for Geriatric Pathology, Tokyo Metropolitan Institute of Gerontology, Tokyo 173-0015, Japan

**Keywords:** H19, cancer stem cell, pancreatic cancer, invasion, integrin

## Abstract

The long non-coding RNA *H19* is highly expressed in several cancers, and the functions of *H19* vary among cancer cell types. Recently, we reported that *H19* contributes to the metastasis of pancreatic ductal adenocarcinoma (PDAC) cells and that inhibition of *H19* reduces metastasis *in vivo*. However, the molecular mechanisms underlying the metastasis-promoting role of *H19* in PDAC cells remain poorly elucidated. In this study, we clarified the mechanisms by which *H19* regulates PDAC metastasis, with a focus on cancer stem cells (CSCs), by using *H19*-overexpressing and knockdown PDAC cells. Whereas the sphere-formation and invasion abilities of PDAC cells depended on *H19* expression levels, other CSC characteristics of the cells, including stemness-marker expression and anticancer-drug resistance, were unaffected by *H19* levels. Furthermore, metalloproteinase activity, a key mediator of invasion, was also independent of *H19* expression. By contrast, *H19* promoted cell adhesion through regulation of integrin and CD24 expression. Notably, the increased adhesion of *H19*-overexpressing cells was blocked by an anti-β1-integrin antibody, and this resulted in the inhibition of sphere formation and invasion. Thus, *H19* plays critical roles in the CSC self-renewal and cell adhesion of PDAC that lead to invasion and metastasis. Our findings suggest that *H19* represents a novel therapeutic target for the metastasis of pancreatic cancer.

## INTRODUCTION

Pancreatic cancer is challenging to diagnose at an early stage and is highly metastatic; consequently, the overall survival rate for patients with pancreatic cancer is only ~8%. Pancreatic cancer is currently the fourth-leading and third-leading cause of cancer death in Japan and USA, respectively [1, 2]. Most pancreatic cancer patients are aged >60 years old, and the aging population is rapidly increasing worldwide. By 2030, pancreatic cancer is expected to become the second-leading cause of cancer-related deaths in USA [3]. A major histological subtype of pancreatic cancer is pancreatic ductal adenocarcinoma (PDAC), which accounts for 90% of all pancreatic cancer cases. Surgical treatment offers the only possible cure for PDAC, but 80% of all PDAC patients are inoperable at diagnosis. Moreover, even after surgery, the 5-year survival rate is only 15%–20%, because of the high metastatic rate and local recurrence of the cancer [4]. Currently, chemotherapies or chemoradiotherapies can reduce tumor size and improve prognosis, but these treatments do not eliminate all PDAC cells in the patients. Therefore, understanding the molecular mechanisms of PDAC carcinogenesis and metastasis could facilitate the identification of potential tumor biomarkers and the development of effective therapeutic strategies against metastasis and recurrence.

According to the central dogma of biology, RNA constitutes the intermediary between DNA and protein. However, recent genome-wide transcriptome studies have revealed that almost the entire human genome is transcribed and produces a plethora of non-coding RNAs (ncRNAs) [5–7]. Long ncRNAs (lncRNAs), which are mRNA-like transcripts containing >200 ribonucleotides, function as critical regulators of gene expression by participating in epigenetic modification, transcriptional control, RNA- processing control, translational control, and posttranslational modification [8]. Accumulating evidence suggests that lncRNAs play crucial roles in carcinogenesis and in the growth and metastasis of various types of cancer, although their detailed mechanisms of action remain unclear [5, 6, 9, 10].

The *H19* gene, located at human chromosome 11p15.5, encodes an imprinted lncRNA. *H19* is transcribed exclusively from the maternal allele, and the gene also generates an oncofetal RNA that is expressed in the developing embryo and in certain types of tumor [11, 12]. Recent evidence indicates that *H19* enhances invasion and metastasis in bladder cancer [13, 14], glioma [15], osteosarcoma [16], acute myeloid leukemia [17], breast cancer [18, 19], non-small cell lung cancer [20], gastric cancer [21], and pancreatic cancer [22], but suppresses the aggressiveness of hepatocellular carcinoma [23] and prostate cancer [24].

We recently reported that *H19* was the highest-expressed ncRNA in PANC-1 lung metastasis-derived human pancreatic cancer cells and that inhibition of *H19* decreased the lung and liver metastases of pancreatic cancer in immunodeficient mice [25]; this finding indicates that *H19* represents a novel candidate for targeted therapy against pancreatic cancer metastasis. However, the molecular mechanisms of *H19* contribution in PDAC cells remain poorly clarified. Therefore, we examined the mechanisms by which *H19* regulates PDAC metastasis, with a focus on cancer stem cells (CSCs), by using PDAC cells in which *H19* was either overexpressed or depleted. Here, we show that *H19* promotes sphere formation, which indicates self-renewal ability, and invasion by regulating integrin and CD24 expression in PDAC cells.

## RESULTS

### *H19* expression in PDAC cells

**Figure 1:**
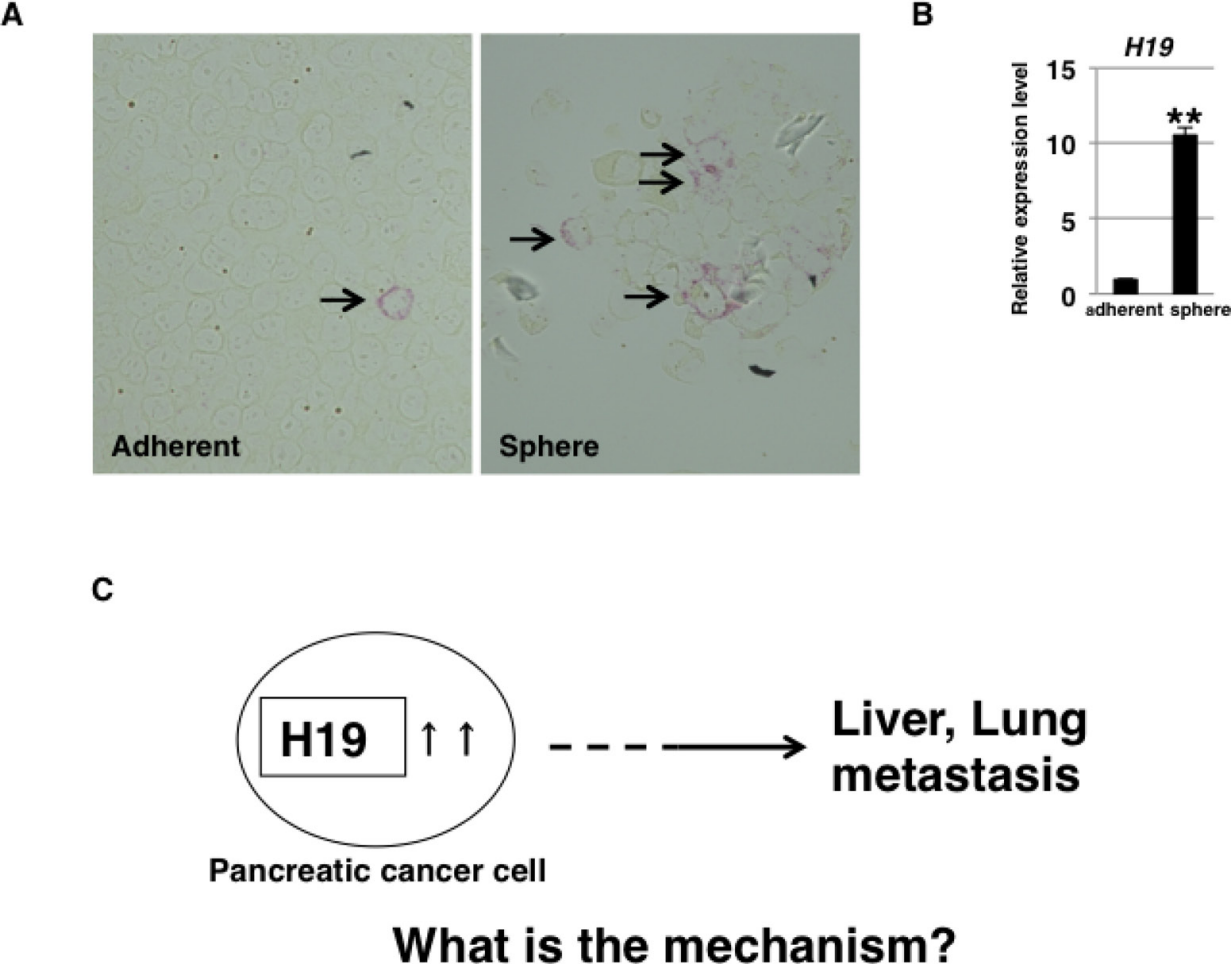
*H19* expression in PDAC cells (**A**) *H19* expression was analyzed by performing *in situ* hybridization in PANC-1 cells. Fewer *H19*-expressing cells were detected among PANC-1 cells cultured under the adherent condition (left) than among PANC-1-cell spheres (right). Original magnification, 400×. (**B**) qRT-PCR analysis of *H19* was performed using cDNA derived from adherent and 3D-cultured PANC-1 cells. ^**^P < 0.01. (**C**) Schematic depiction of the question addressed in this study. Results are presented as means ± SD from three independent experiments.

To determine whether *H19* is expressed heterogeneously or homogeneously in human PDAC cells, we examined *H19* expression in PANC-1 cells by using a highly sensitive *in situ* hybridization technique. Under the adherent-culture condition, PANC-1 cells showed heterogeneous *H19* expression and the presence of small populations of *H19-*expressing cells (Figure 1A, left panel, arrow). In a previous report [26], we showed that 3D-culture conditions induce CSC-like populations in PANC-1 cells. Here, at 7 days after sphere formation under 3D-culture conditions, higher *H19* expression was detected among the sphere cells than in cells cultured under the adherent-culture condition (Figure 1B). Numerous *H19-*expressing PANC-1 cells were detected in spheres by using *in situ* hybridization (Figure 1A, right panel, arrow). These results suggest that *H19* is expressed in CSC-like cells among PANC-1 cells. CSCs are responsible for tumor initiation, growth, and even metastasis [27]. We previously showed that *H19* contributes to liver and lung metastases in PANC-1 cells [25]. Thus, we hypothesized that a correlation exists between *H19* and CSCs, and we examined the mechanisms by which *H19* affects CSC phenotypes (Figure 1C).

### *H19* contributes to sphere formation in PDAC cells

**Figure 2:**
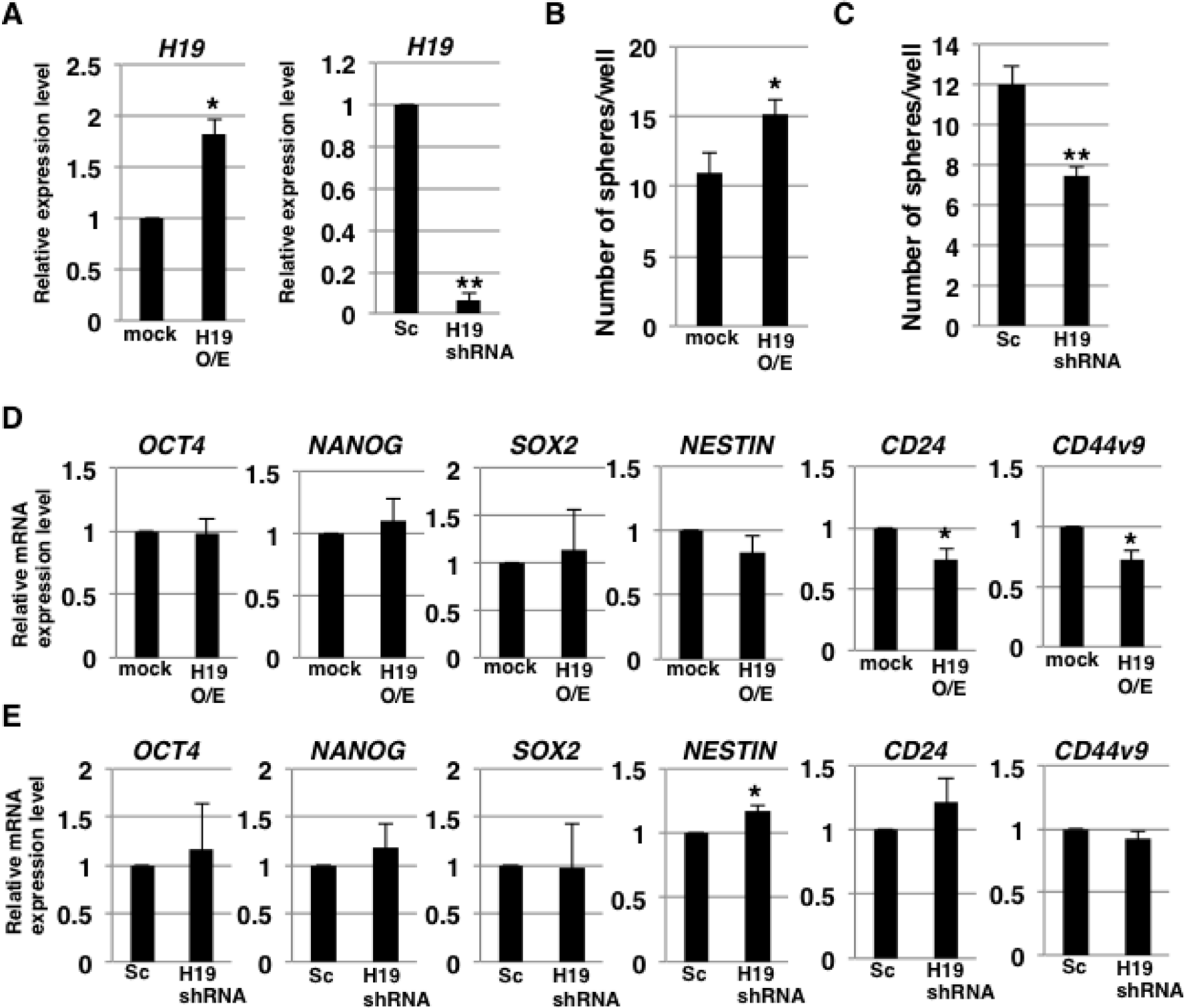
*H19* contributes to sphere formation in PDAC cells (**A**) qRT-PCR analysis of *H19* was performed using cDNA derived from mock and *H19*-overexpressing cells or Sc and *H19*-knockdown cells. Results are normalized relative to the values obtained for mock or Sc cells (value = 1). ^*^P < 0.05, ^**^P < 0.01. (**B** and **C**) Results of sphere-formation assays showing increased and decreased sphere formation by, respectively, *H19*-overexpressing cells (B) and *H19*-knockdown cells (C). ^*^P < 0.05, ^**^P < 0.01. (**D** and **E**) qRT-PCR analysis of stemness markers was performed using cDNA derived from mock and *H19*-overexpressing cells (D) or Sc and *H19*-knockdown cells (E). Results are normalized relative to the values obtained for mock or Sc cells (value = 1). ^*^P < 0.05. Results are presented as means ± SD from three independent experiments.

To clarify the involvement of *H19* in the development of CSC characteristics, we examined self-renewal ability and CSC-marker expression in *H19*-overexpressing and -knockdown PDAC cells by performing sphere-formation assays and stemness-marker analysis. Quantitative reverse transcription-polymerase chain reaction (qRT-PCR) analysis confirmed increased and decreased *H19* expression in *H19*-overexpressing and -knockdown PANC-1 cells, respectively (Figure 2A). The results of sphere-formation assays showed that relative to the control, *H19*-overexpressing and -knockdown cells exhibited, respectively, higher sphere formation (Figure 2B) and lower sphere formation (Figure 2C). Furthermore, qRT-PCR analysis revealed that 2 out of 6 examined stemness-markers (*CD24* and *CD44v9*) were expressed at lower levels in *H19*-overexpressing cells than in mock cells (Figure 2D), whereas, 1 of the 6 markers (*NESTIN*) was expressed at a higher level in *H19*-knockdown cells than in scrambled-sequence short-hairpin (sh) RNA-transfected (Sc) cells (Figure 2E). These results indicate that *H19* promotes sphere-formation but is not clearly involved in stemness-marker expression in PDAC cells.

**Figure 3:**
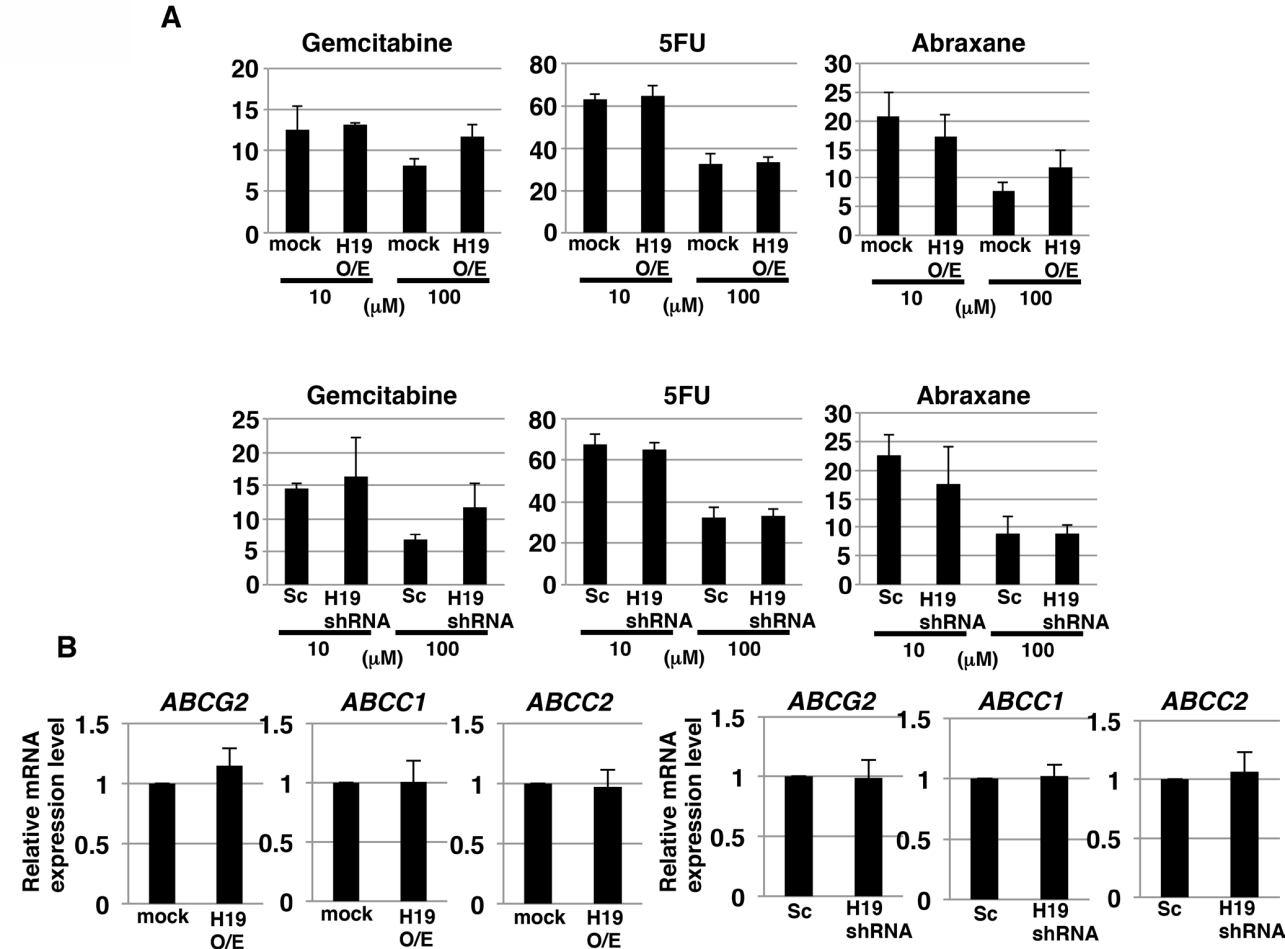
*H19* does not contribute to anticancer-drug resistance in PDAC cells (**A**) ATP assay results showing the resistance of mock and *H19*-overexpressing cells or Sc and *H19*-knockdown cells against three drugs tested at 10 and 100 μM: gemcitabine, 5-FU, and abraxane. (**B**) qRT-PCR analysis of transporter expression was performed using cDNA derived from mock and *H19*-overexpressing cells or Sc and *H19*-knockdown cells. Results are presented as means ± SD from three independent experiments.

CSCs possess an effective efflux pathway for anticancer drugs. Thus, we next examined whether *H19* contributes to anticancer-drug resistance in PDAC cells. We tested three commonly used anti-pancreatic cancer drugs, gemcitabine, 5-FU, and abraxane. Survival rates of the cells after addition of gemcitabine, 5-FU, and abraxane (all at 100 μM) were approximately 10%, 30%, and 10%, respectively (Figure 3A). The survival rates did not differ in a statistically significant manner between mock and *H19*-overexpressing cells or between Sc and *H19*-knockdown cells, after treatment with the anticancer drugs at either 10 or 100 μM (Figure 3A). Furthermore, we examined the gene-expression levels of three potential anticancer drug transporters: qRT-PCR analysis revealed that the expression of *ABCG2*, *ABCC1*, and *ABCC2* was not significantly different between mock and *H19*-overexpressing cells, or between Sc and *H19*-knockdown cells (Figure 3B). These results indicate that *H19* is not involved in regulating the expression of anticancer drug transporters and the resistance toward anticancer drugs in PDAC cells.

### *H19* promotes invasion in PDAC cells

**Figure 4:**
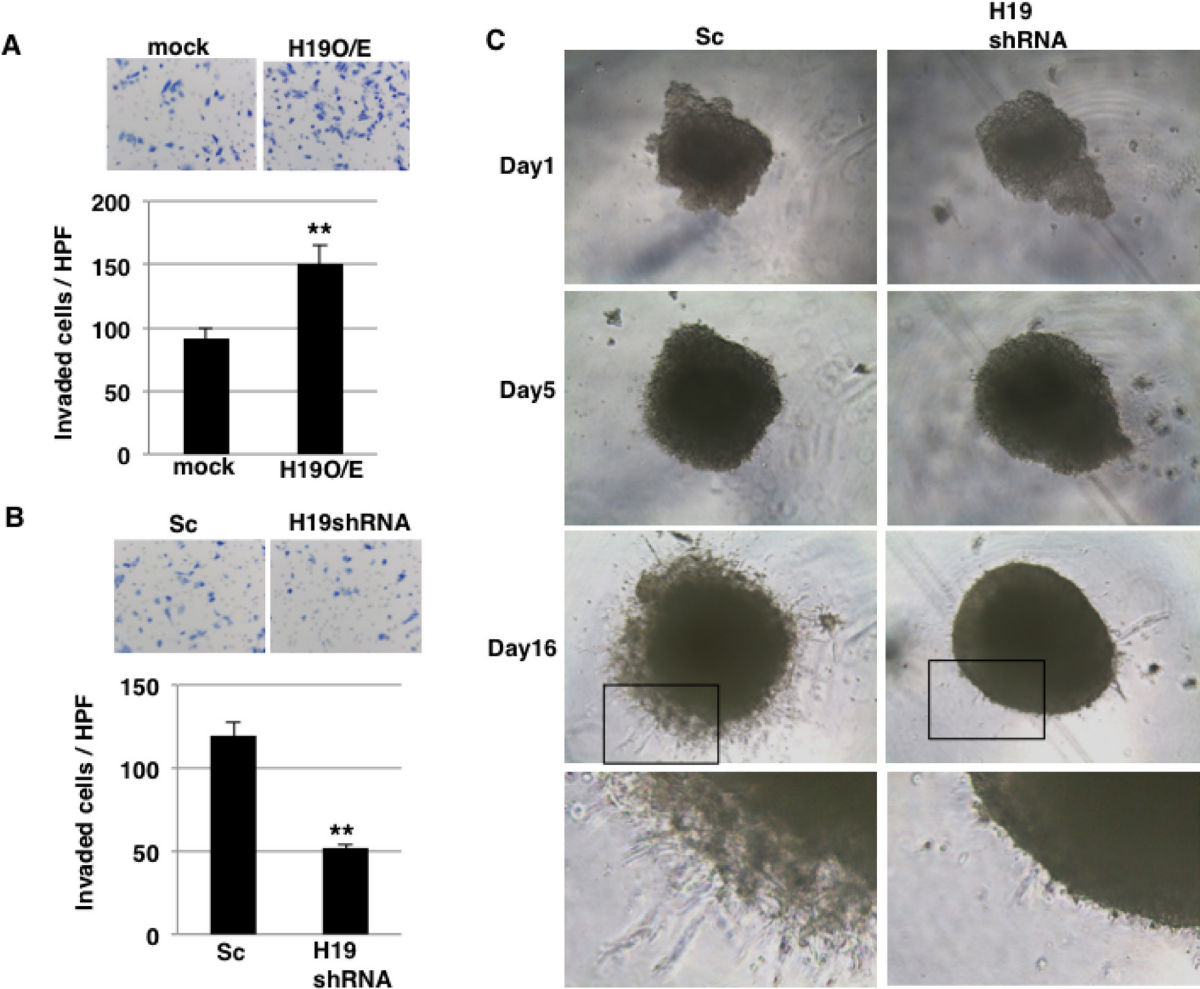
*H19* promotes invasion in PDAC cells (**A** and **B**) Matrigel invasion assays were performed using mock and *H19*-overexpressing cells (A) or Sc and *H19*-knockdown cells (B). ^**^P < 0.01. (**C**) 3D invasion assays were performed using Sc and *H19*-knockdown cells. Representative images from Days 1, 5, and 16 are shown. The high-magnification images show that typical invasive cells were more numerous among Sc cells than *H19*-knockdown cells.

In our previous report [25], we demonstrated that *H19* contributes to liver and lung metastases in PDAC cells. During the metastatic cascade, invasion into the surrounding stroma is a key step, and we thus investigated whether *H19* functions in the invasion process. In invasion assays performed using inserts coated with Matrigel, which mimics the *in vivo* basement membrane, a larger number of *H19*-overexpressing cells than mock cells invaded through the pores of the insert membrane (Figure 4A). By contrast, *H19*-knockdown cells exhibited lower invasion than Sc cells (Figure 4B). Under 3D-culture conditions, CSC-like populations are enriched, and we showed that *H19* was highly expressed in sphere cells (Figure 1B). Therefore, to clarify the contribution of *H19* in sphere cells, we performed 3D invasion assays, which revealed that in the 3D invasion, Sc sphere cells exhibited the typical starburst invasion pattern to a greater extent than did *H19*-knockdown sphere cells at Day 16 (Figure 4C). These results indicate that *H19* contributes to invasion in PDAC cells.

**Figure 5:**
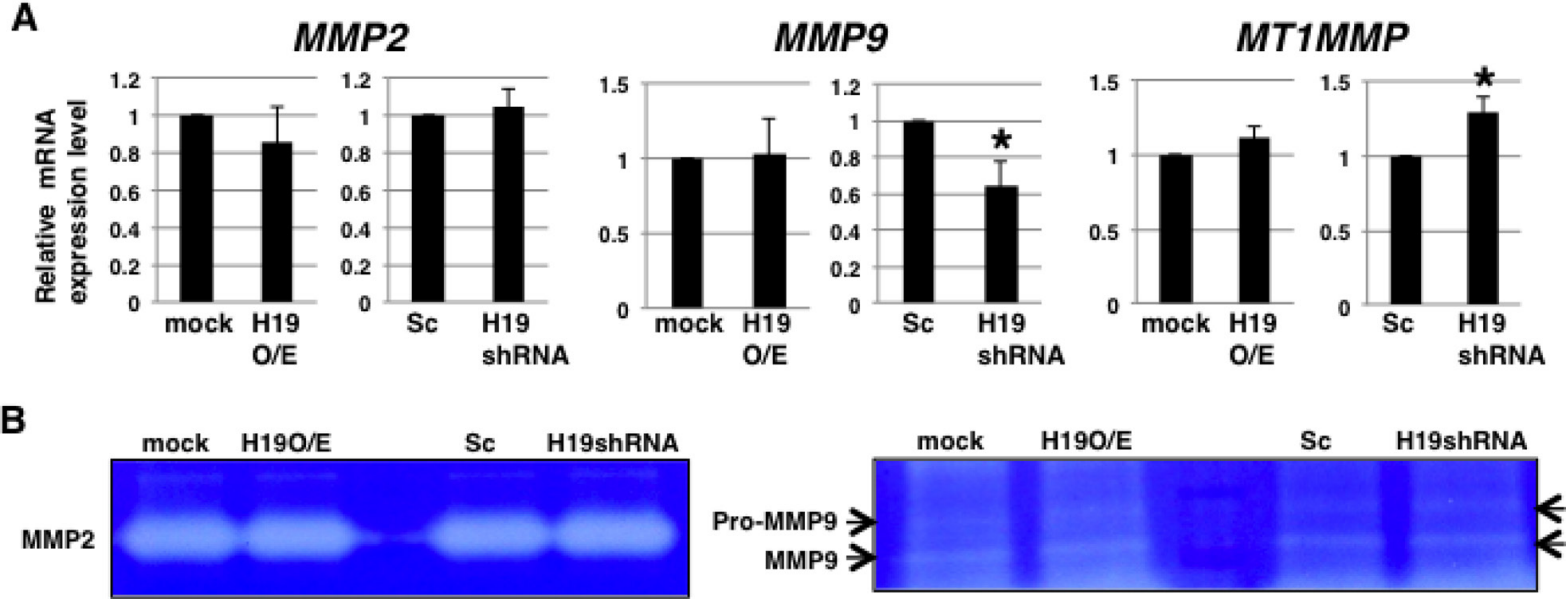
*H19* does not contribute to MMP activity in PDAC cells (**A**) qRT-PCR analysis of MMPs was performed using cDNA derived from mock and *H19*-overexpressing cells or Sc and *H19*-knockdown cells. Results are presented as means ± SD from three independent experiments. ^*^P < 0.05. (**B**) Gelatin zymography analyses were performed using culture supernatants from mock and *H19*-overexpressing cells or Sc and *H19*-knockdown cells. Representative results from three independent experiments are shown.

Highly elevated production of matrix metalloproteinases (MMPs) is recognized as a key feature of invasion [28]. Thus, we hypothesized that *H19* might promote MMP expression and activity. However, qRT-PCR analysis revealed that the expression levels of *MMP2*, *MMP9*, and *MT1MMP*, which are known to be expressed in PDAC cells [29, 30], were not substantially different between *H19*-overexpressing cells and mock cells (Figure 5A), although relative to Sc cells, *H19*-knockdown cells showed *MMP9* downregulation and *MT1MMP* upregulation (Figure 5A). Moreover, the collagenase activity of MMP2 and MMP9 did not differ markedly between mock and *H19*-overexpressing cells, or between Sc and *H19*-knockdown cells (Figure 5B). These results suggest that *H19* is not involved in the regulation of MMP expression and activity in PDAC cells.

### *H19* contributes to cell adhesion through regulation of CD24 and integrin expression in PDAC cells

**Figure 6:**
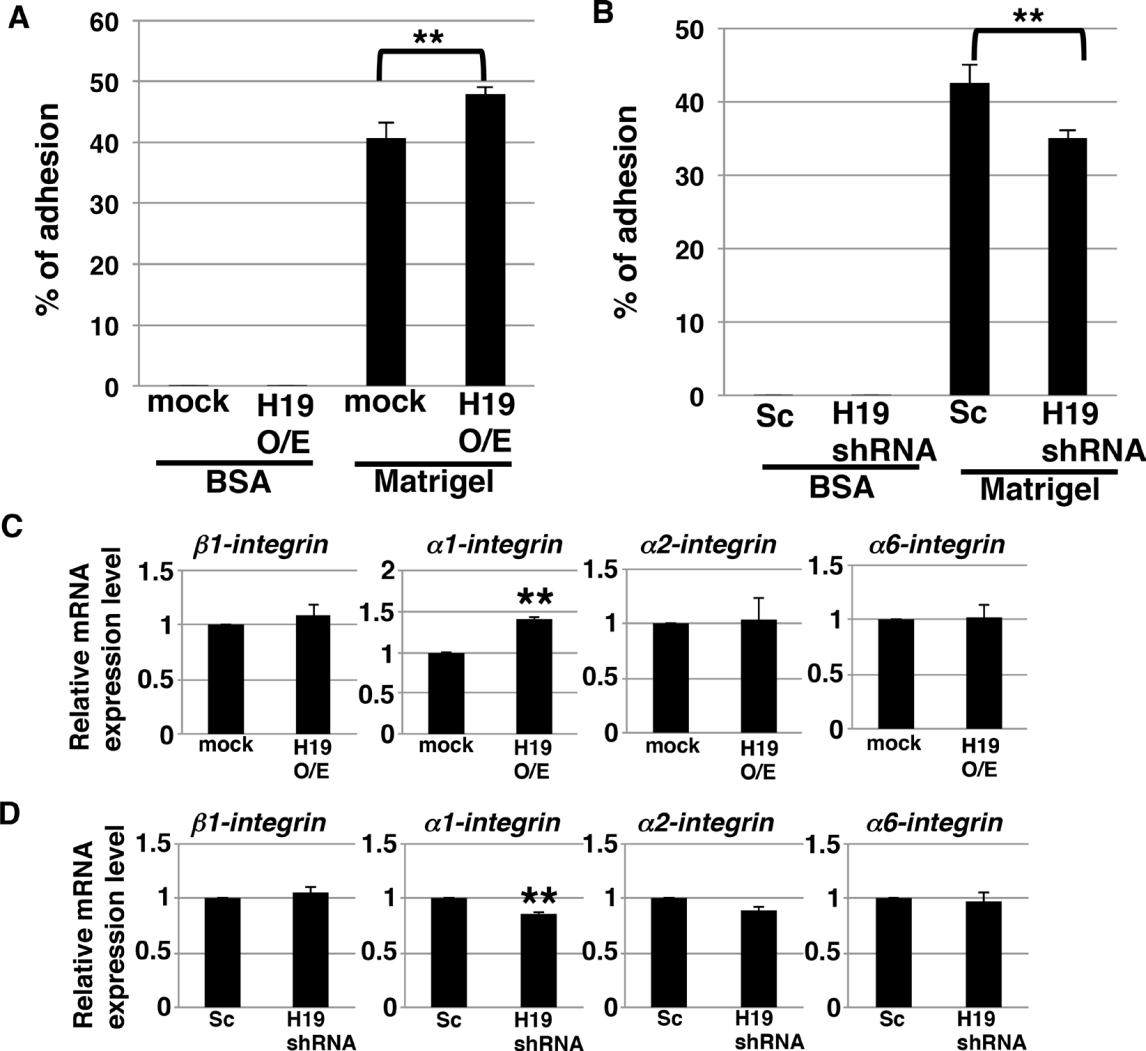

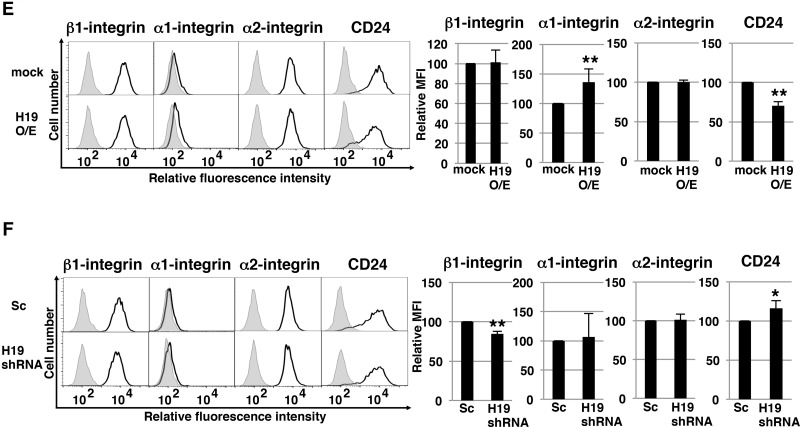
*H19* contributes to cell adhesion through regulation of CD24 and integrin expression in PDAC cells (**A** and **B**) Adhesion assays were performed using mock and *H19*-overexpressing cells (A) or Sc and *H19*-knockdown cells (B). Each cell type exhibited negligible adhesion to BSA. ^**^P < 0.01. (**C** and **D**) qRT-PCR analysis of integrins was performed using cDNA derived from mock and *H19*-overexpressing cells (**C**) or Sc and *H19*-knockdown cells (**D**). Results are presented as means ± SD from three independent experiments. ^**^P < 0.01. (**E** and **F**) Flow-cytometry analysis of integrin and CD24 levels in mock and *H19*-overexpressing cells (E) or Sc and *H19*-knockdown cells (F). Controls are indicated by thin lines with gray color. MFIs relative to mock or Sc cells are shown on the right side. Results are presented as means ± SD from three independent experiments. ^*^P < 0.05, ^**^P < 0.01.

During the process of invasion, a critical event is the adhesion of cancer cells to the extracellular matrix [31]. We investigated whether *H19* contributes to this cell-to-matrix adhesion step. Adhesion assays on Matrigel revealed that *H19*-overexpressing cells exhibited a statistically significant increase in adhesiveness (~50% adherent cells, versus ~40% in the case of mock cells) (Figure 6A), whereas *H19*-knockdown cells exhibited a significant decrease in adhesiveness (~35% adherent cells, versus ~40% in the case of Sc cells) (Figure 6B). These results indicate that *H19* supports cell-matrix adhesion. Integrins, which mediate the crosstalk between tumor cells and basement-membrane proteins, are major cell-surface receptors and play a critical role in cell adhesion, invasion, and metastasis induction [32–34]. We therefore investigated whether *H19* promotes cell adhesion through regulation of integrins. qRT-PCR results showed that, while α*1-integrin* was upregulated in *H19*-overexpressing cells (Figure 6C), it was downregulated in *H19*-knockdown cells (Figure 6D), which suggests that *H19* positively regulates the expression of α*1-integrin*. Furthermore, we examined the cell-surface expression of integrins and that of the epithelial marker CD24. Fluorescence-activated cell sorting (FACS) analysis revealed that α1-integrin expression was low in mock cells, but markedly increased in *H19*-overexpressing cells, and that CD24 expression was reduced in *H19*-overexpressing cells (Figure 6E). This suggested that *H19*-overexpressing cells present metastatic mesenchymal phenotypes. By contrast, *H19*-knockdown cells exhibited a substantial reduction of β1-integrin expression but elevated expression of CD24 relative to Sc cells (Figure 6F), suggesting that *H19*-knockdown cells are more epithelial-cell-like and less metastatic than Sc cells. Taken together, these results support our proposal that *H19* contributes to cell adhesion by regulating both the transition of epithelial cancer cells to a mesenchymal state and integrin expression.

### Blocking β1-integrin inhibits sphere formation and invasion in *H19*-overexpressing cells

**Figure 7:**
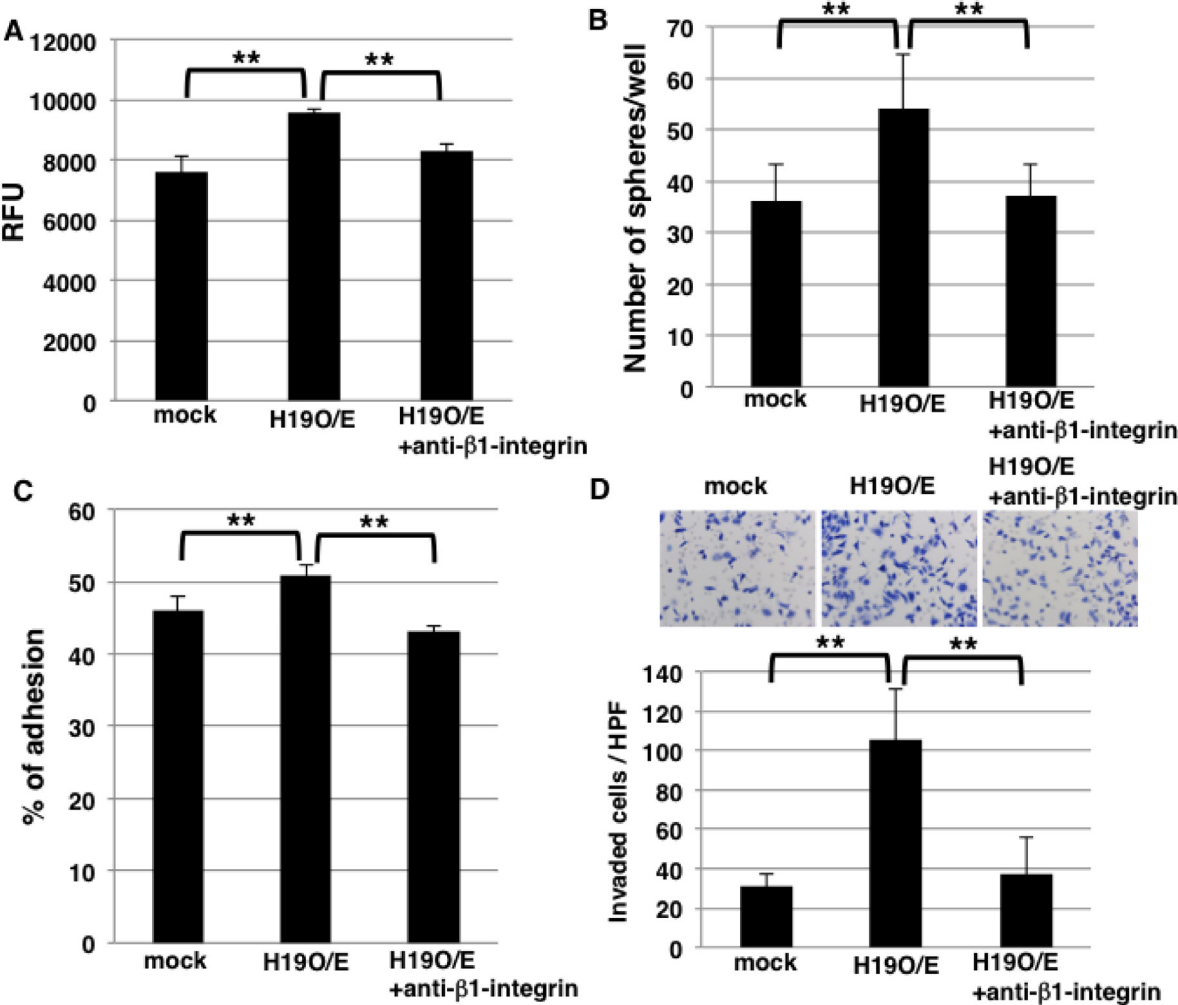
Blocking of β1-integrin inhibits sphere formation and invasion in *H19*-overexpressing cells (**A**) Cell-cell adhesion assays were performed on mock and *H19*-overexpressing cells, with or without anti-β1-integrin antibody treatment. Adherent cells were indicated by RFU (relative fluorescence units). ^**^P < 0.01. (**B**) Results of sphere-formation assays showing that the formation of an increased number of spheres by *H19*-overexpressing cells was inhibited by anti-β1-integrin antibody treatment. ^**^P < 0.01. (**C**) Adhesion assays were performed on mock and *H19*-overexpressing cells, with or without anti-β1-integrin antibody treatment. ^**^P < 0.01. (**D**) Matrigel invasion assays were performed on mock and *H19*-overexpressing cells, with or without anti-β1-integrin antibody treatment. ^**^P < 0.01.

Sphere formation was higher in *H19*-overexpressing cells than mock cells (Figure 2B), but the underlying mechanism is unknown. Because our results showed that *H19* promotes cell adhesion by regulating integrin expression (Figure 6), we hypothesized that the increased sphere formation by *H19*-overexpressing cells depends on integrin-mediated cell adhesion. To test this, we included an anti-β1-integrin blocking antibody in cell-cell adhesion assays and sphere-formation assays to supress integrin-mediated adhesion; cell-cell adhesion assays revealed that the increased adhesion exhibited by *H19*-overexpressing cells was inhibited by the blocking antibody (Figure 7A), indicating that the enhanced cell-cell adhesiveness was dependent on β1-integrin. Furthermore, the blocking antibody inhibited the increased sphere formation observed with *H19*-overexpressing cells (Figure 7B), which indicates that *H19* contributes to sphere formation through integrin-mediated cell adhesion.

Lastly, to clarify whether *H19* positively regulates invasion by modulating integrin expression, we quantified invasion by using *H19*-overexpressing cells treated with the anti-β1-integrin blocking antibody. Adhesion assays on Matrigel revealed that the increased adhesion exhibited by *H19*-overexpressing cells was inhibited by the blocking antibody (Figure 7C), which indicates that the enhanced adhesiveness of these cells depends on β1-integrin. Furthermore, the increased invasion shown by *H19*-overexpressing cells was also inhibited by the anti-β1-integrin blocking antibody (Figure 7D). These results indicate that *H19* contributes to invasion through the regulation of β1-integrin-mediated cell adhesion.

### EMT induction in *H19*-knockdown PDAC cells

**Figure 8:**
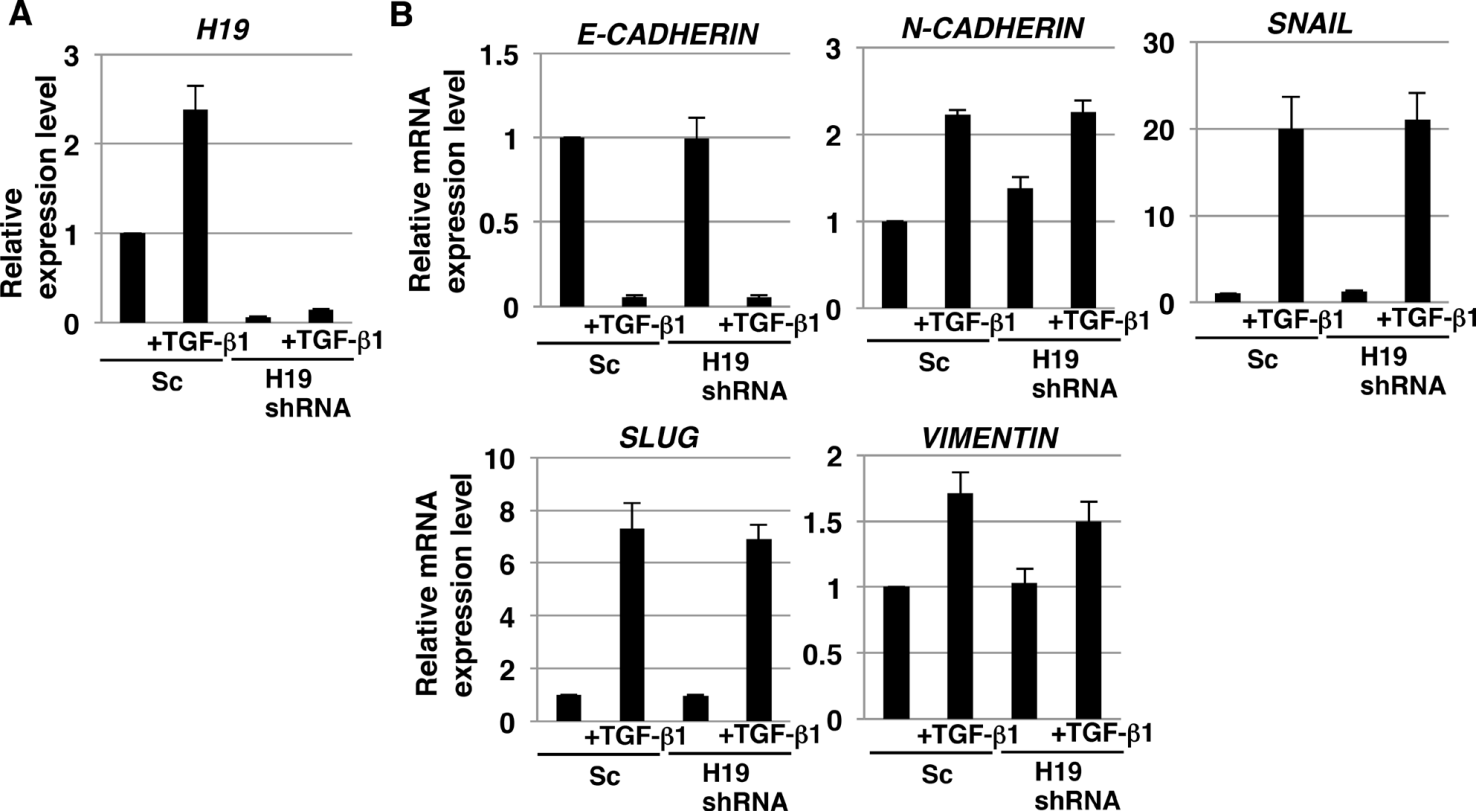
EMT induction in H19-knockdown PDAC cells qRT-PCR analysis of *H19* (**A**) and EMT markers (**B**) performed using cDNA derived from Sc and *H19*-knockdown cells after EMT induction. Results are normalized relative to values obtained for non-treated Sc cells (value = 1). Results are presented as means ± SD from three independent experiments.

*H19* has been reported to play a role in epithelial to mesenchymal transition (EMT), which is the initial key process required for tumor metastasis in certain cancer cells [35–37]. However, no study thus far has clarified the contribution of *H19* to the EMT of PDAC cells. As discussed in the preceding sections, our results demonstrated *H19* involvement in EMT-like behaviors such as cell adhesion and invasion. We therefore investigated whether *H19* plays a role in the EMT of PDAC cells. qRT-PCR analysis revealed that *H19* expression was increased in Sc cells, but not *H19*-knockdown cells, after treatment with even comparatively lower concentrations of transforming growth factor-β1 (TGF-β1) (2 ng/mL) (Figure 8A), and EMT-marker expression was confirmed to be substantially altered in PANC-1 cells treated with TGF-β1 at various concentrations (0.5–4 ng/mL) (unpublished data); this suggested the involvement of *H19* in EMT. However, the decrease in *E-CADHERIN* and increase in other EMT markers were comparable in Sc and *H19*-knockdown cells (Figure 8B). Based on these results, we propose two reasons for the lack of a major contribution of *H19* in the EMT of PDAC cells: one, TGF-β1 signaling is functionally independent from *H19* contribution; and two, the effect of TGF-β1 signaling predominates when compared with the effect of *H19* in PDAC.

### *H19*-knockdown effect on PK-59 PDAC cells

**Figure 9:**
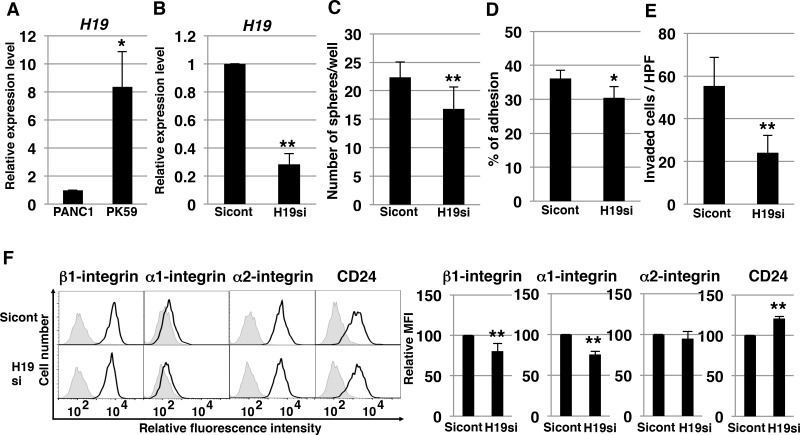
*H19*-knockdown effect on PK-59 PDAC cells (**A**) qRT-PCR analysis of *H19* was performed using cDNA derived from PANC-1 and PK-59 cells. ^*^P < 0.05. (**B**) qRT-PCR analysis of *H19* was performed using cDNA derived from Sicont and *H19*-knockdown (*H19*si) PK-59 cells. Results are normalized relative to the values obtained for sicont cells (value = 1). ^**^P < 0.01. (**C**) Results of sphere-formation assays showing decreased sphere formation by *H19*-knockdown (*H19*si) PK-59 cells. ^**^P < 0.01. (**D**) Adhesion assays were performed on Sicont and *H19*-knockdown (*H19*si) PK-59 cells. ^*^P < 0.05. (**E**) Matrigel invasion assays were performed on Sicont and *H19*-knockdown (*H19*si) PK-59 cells. ^**^P < 0.01. (**F**) Flow-cytometry analysis of integrin and CD24 levels in Sicont and *H19*-knockdown (*H19*si) PK-59 cells. Controls are indicated by thin lines with gray color. MFIs relative to Sicont cells are shown on the right side. Results are presented as means ± SD from three independent experiments. ^**^P < 0.01.

As shown above, from among the available PDAC cell lines we used PANC-1 to demonstrate the molecular mechanisms of *H19*. We also tested the mechanism of *H19* in other PDAC cells. Among human PDAC cell lines, we found that *H19* expression in PK-59 was about 8 times higher than in PANC-1 cells (Figure 9A). Following knockdown of *H19* in PK-59 cells, we performed several experiments. qRT-PCR analysis confirmed decreased *H19* expression in *H19*-knockdown PK-59 cells (Figure 9B). In these cells, sphere formation was significantly decreased (Figure 9C), as were cell adhesion to Matrigel and invasion (Figure 9D and 9E). Furthermore, *H19*-knockdown PK-59 cells exhibited a substantial reduction of β1- and α1-integrin expression but elevated expression of CD24 relative to Sicont cells (Figure 9F). Thus, the majority of these results were consistent with the results of PANC-1 cells, indicating that *H19* contributes to sphere formation and invasion by regulating integrin and CD24 expression in PDAC cells.

## DISCUSSION

*H19* involvement in invasion and/or metastasis has been demonstrated in several cancers. We previously reported that in pancreatic cancer, inhibition of *H19* reduced cancer metastasis *in vivo* [25], which demonstrated that *H19* promotes the metastasis of PDAC cells. However, the molecular mechanisms underlying *H19* contribution to PDAC-cell metastasis have remained unknown thus far. In this study, we showed that *H19* contributes to sphere formation in PDAC cells, but not to stemness-marker expression and anticancer-drug resistance, and further promotes invasion by regulating integrin and CD24 expression in PDAC cells.

To date, several studies have reported a correlation between *H19* and stemness. In normal prostate cells, *H19* promotes the expression of stemness markers [38]. Furthermore, *H19* supports sphere formation in breast cancer cells [19, 39], as well as transporter expression and anti-cancer-drug resistance in hepatocellular carcinoma cells [40]. Here, we showed that in PDAC cells, *H19* promotes sphere formation, but not stemness-marker expression or transporter expression and anti-cancer-drug resistance. Although further studies are required to clarify this matter, we speculate that the involvement of *H19* differs among cancer cell types due to the existing balance in the expression of *H19*, its target gene and proteins, and micro-RNAs. The results of *in situ* hybridization and qRT-PCR analysis suggested that *H19* is highly expressed in the PDAC CSC-like cells (Figure 1). Therefore, clarification of the specificity of *H19* expression in CSCs could lead to the identification of a novel CSC marker in PDAC.

The involvement of *H19* in cell adhesion and integrin expression in cancer cells has been reported in only a few previous studies. In endometrial carcinoma, *H19* regulates the expression of the β5, β3, and α4 integrins, which presumably leads to enhanced motility and increased invasive potential of the cells [41]. In this study, we showed that *H19* is involved in cell adhesion and integrin expression in PDAC cells. However, no currently-available evidence indicates that *H19* contributes directly to the regulation of integrin expression at the mRNA or protein level. Therefore, to clarify the correlation between *H19* and integrin expression, further studies, such as epigenomic analyses, are required.

Enhanced CD24 expression in various cancers, including PDAC, has been reported, and CD24 expression correlates with malignancy [42, 43]. Among PDAC cells, the cells that expressed CD24, CD44, and epithelial-specific antigen on their surface were identified as putative CSCs [44]. CD24 expression also regulates cell motility and invasion [45, 46] and contributes to the maintenance of the epithelial phenotype of PDAC cells [47]. Here, we showed for the first time that increased *H19* expression leads to a reduction of cell-surface CD24, and that downregulation of *H19* helps maintain cell-surface CD24 expression. Therefore, we propose that *H19* contributes to cell invasion by regulating CD24 expression. In a previous report [48], CD24 expression was shown to correlate with high invasive ability in PDAC cells. By contrast, we showed that *H19*-overexpressing PDAC cells, which are highly invasive, exhibited a reduction of CD24 expression. Therefore, further investigation is necessary to clarify the functional effect of *H19* on CD24 expression and on invasiveness through regulation of CD24 expression. In breast cancer, CD24 is negative in CSCs, and CD24-negative cells are tumorigenic and invasive [49, 50]. From our results, we speculate that the overexpression of *H19* in malignant CSCs of breast cancer contributes to reduction in CD24 expression and causes the invasive phenotype. In contrast, *H19* expression is normally lowered in CD24-positive CSCs in PDACs, but is induced during transformation from the epithelial to the mesenchymal state, and CD24 is reduced in the cells which change to the invasive phenotype.

**Figure 10:**
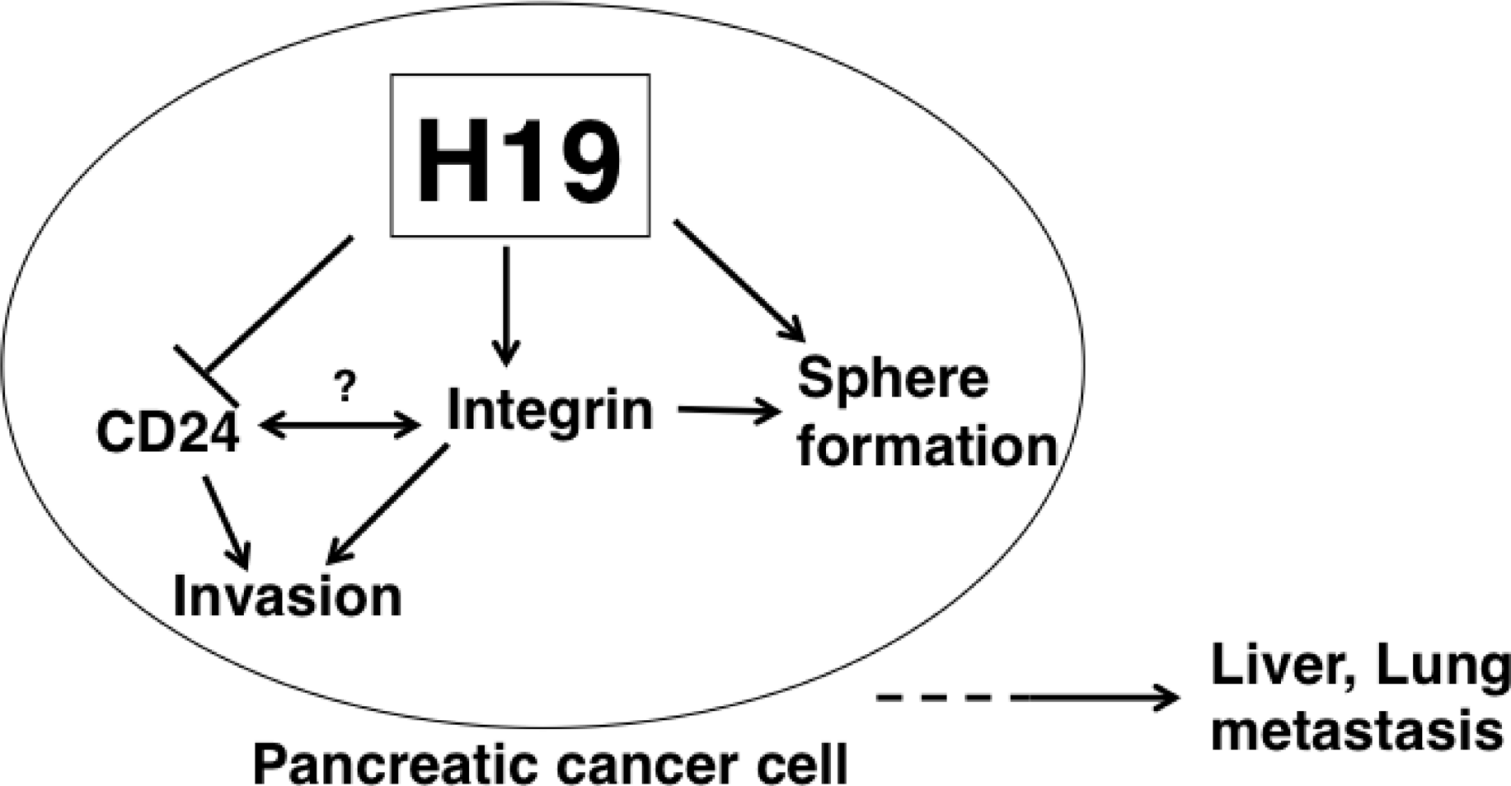
Model depicting *H19* role in promoting PDAC-cell metastasis In *H19-*expressing PDAC cells, sphere formation and cell invasion are enhanced. *H19* is suggested to directly or indirectly contribute to CD24 and integrin expression. Interactions between CD24 and integrin are possible according to previously reported results [51]. *H19*-mediated downregulation of CD24 and upregulation of integrin are considered to induce an EMT-like phenotype, such as the enhanced cell adhesion resulting in a high invasive ability of PDAC cells. Collectively, our findings suggest that induction of the EMT-like phenotype by *H19* leads to liver or lung metastasis.

In conclusion, we have demonstrated that *H19* contributes to sphere formation and invasion by regulating integrin and CD24 expression in PDAC cells, thus presumably leading to liver and lung metastasis (Figure 10). Therefore, *H19* expression in PDAC is correlated with malignancy potential, and elimination of *H19*-expressing cells might represent a novel therapeutic option for PDAC. Further studies are required to clarify the characteristics of *H19*-expressing cells in PDAC, as well as to develop agents that eliminate *H19*-expressing cells for the treatment of PDAC. Specifically, the cell-surface characteristics of *H19*-expressing cells could be useful for the detection and elimination of *H19*-expressing cells. For example, a specific antigen present in *H19*-expressing cells might serve as a novel biomarker of malignant CSCs and could be applied to the development of therapeutic-antibody drugs and photoimmunotherapy in PDAC.

## MATERIALS AND METHODS

### Cell culture

The human PDAC cell lines PANC-1 and PK-59 were obtained from the Cell Resource Center for Biomedical Research, Institute of Development, Aging and Cancer, Tohoku University (Sendai, Japan). PANC-1 and PK-59 cells were grown in growth medium (RPMI 1640 medium containing 10% fetal bovine serum) at 37°C under a humidified 5% CO_2_ atmosphere. For EMT induction, cells were cultured for 72 h in growth medium containing 2 ng/mL TGF-β1 (Peprotech, Rocky Hill, NJ, USA). For 3D culture, cells (3.0 × 10^3^ cells/well) in growth medium were plated in 96-well ultra-low attachment plates (Thermo Fisher Scientific, Waltham, MA, USA). After 7 days, the spheres were aspirated using micropipettes and placed in microcentrifuge tubes for use in further experiments.

### Generation of stable H19-overexpressing and *H19*-shRNA-expressing PANC-1 cells

The *H19* full-length cDNA (2.3 kb) cloned into pcDNA3.1(−) vector (a generous gift from Dr. Eric Adriaenssens, Science and Technology, Lille 1 University, France) and empty vector were transfected into PANC-1 cells by using the FuGENE HD transfection reagent (Roche Diagnostics, Mannheim, Germany) according to the manufacturer's instructions. Briefly, 2 × 10^5^ cells/mL were transfected with 5 μg of DNA by using FuGENE HD and then the cells were passaged and cultured with 1 mg/mL geneticin (Sigma-Aldrich Corporation, St. Louis, MO, USA) [25].

To construct the expression vector for human *H19* shRNA, a DNA fragment flanked by *Bam*HI and *Hind*III sites and containing the sense target sequence for *H19* (5′-TCA TCA GCC CAA CAT CAA A-3′), the hairpin-loop sequence (5′-TAG TGC TCC TGG TTG-3′), and the antisense target sequence was synthesized and inserted into the pBAsi-hU6 Neo DNA vector. Similarly, a scrambled sequence (5′-TCT TAA TCG CGT ATA AGG C-3′) was used to construct the sham vector that served as the negative control. The *H19* shRNA and scrambled shRNA vectors were transfected as described in the preceding paragraph.

### Transfection of siRNA targeting *H19*

We used siRNA (5′-CCC UCU AGC UUG GAA AUG ATT-3′) targeting *H19* RNA as shown in previous our report [25]. The cells were plated at a density of 2 × 10^5^ cells in 35-mm dishes and transfected 24 hours later with 5 nM of either siRNAs targeting *H19* or Silencer negative control siRNA (sicont) using Lipofectamine™ RNAiMAX Transfection Reagent (Thermo Fisher Scientific) according to the manufacturer's protocol. Each experiment was performed 72 hours after transfection.

### Branched DNA *in situ* hybridization

Branched DNA *in situ* hybridization was performed according to the protocol provided with the ViewRNA ISH Tissue 1-Plex Assay Kit (Affymetrix, Inc., Santa Clara, CA, USA) [18, 25]. Briefly, sections of PANC-1-cell blocks were deparaffinized, boiled in Pretreatment Solution (1:100) for 10 min at 90–100°C, and digested with protease (1:100) for 10 min at 40°C, and then hybridized (3 h, 40°C) with the H19 probe set, GAPD probe set (positive control), or *Escherichia coli* K12 dapB probe set (negative control) (each diluted 1:40). After washing, the sections were hybridized with PreAmplifier probe (1:100) for 25 min at 40°C, washed, hybridized with Amplifier probe (1:100) for 15 min at 40°C, washed, and, lastly, hybridized with Label Probe conjugated to alkaline phosphatase (1:1000) for 15 min at 40°C. After washing, the sections were incubated with Fast Red substrate. Phase-contrast images were obtained using a BX-9000 microscope (KEYENCE, Osaka, Japan).

### FACS analysis

Cells were harvested after treatment with Accutase^®^ cell-detachment solution (Merck Millipore, Billerica, MA, USA), and the dissociated single cells were incubated with primary antibodies or fluorescein isothiocyanate (FITC)-conjugated antibodies diluted in FACS buffer (0.5% [w/v] bovine serum albumin [BSA] and 0.1% [w/v] sodium azide in PBS) for 30 min on ice. After washing, the cell suspension was incubated with Alexa Fluor^®^ 488-conjugated secondary antibodies (Molecular Probes, Eugene, OR, USA) diluted in FACS buffer for 30 min on ice. Cell sorting and analysis were performed using a FACSAria™ Cell Sorter (Becton Dickinson, Franklin Lakes, NJ, USA). We used the following FITC-conjugated antibodies and primary antibodies: FITC-conjugated isotype control (Becton Dickinson), FITC-conjugated anti-α1-integrin (BioLegend, San Diego, CA, USA), FITC-conjugated anti-α2-integrin (BioLegend), anti-β1-integrin (Abcam, Cambridge, UK), and anti-CD24 (Santa Cruz Biotechnology, Dallas, TX, USA). Mean fluorescence intensities (MFIs) were obtained after subtracting the intensities of the controls.

### qRT-PCR

Total RNA was isolated from cells by using an RNeasy plus mini kit (QIAGEN, Hilden, Germany) and subsequently reverse-transcribed using a ReverTra Ace^®^ qPCR RT Kit (Toyobo, Osaka, Japan). Real-time PCR was performed using a Power Sybr^®^ Green kit (Applied Biosystems, Foster City, CA, USA) and a StepOnePlus™ real-time PCR system (Applied Biosystems). As an internal control, β-actin was amplified. qRT-PCR analysis for *H19* was performed using TaqMan Fast Universal PCR Master Mix (Life Technologies Corporation Carlsbad, CA, USA) and TaqMan Gene Expression Assays (Life Technologies), with 18 S rRNA serving as an internal control. Table 1 lists the primer sets used for real-time PCR.

**Table 1:** Primer sets used for real-time PCR

Gene	Forward primer	Reverse primer
NANOG	CCAAAGGCAAACAACCCACTT	CGGGACCTTGTCTTCCTTTTT
SOX2	TGCGAGCGCTGCACAT	TCATGAGCGTCTTGGTTTTCC
OCT4	GGAGGAAGCTGACAACAATGAAA	GGCCTGCACGAGGGTTT
NESTIN	TCCTGCTGTAGATGCAGAGATCAG	ACCCTGTGTCTGGAGCAGAGA
CD24	TCCAACTAATGCCACCACCAA	GACCACGAAGAGACTGGCTGTT
CD44v9	AGCAGAGTAATTCTCAGAGCTT	TGCTTGATGTCAGAGTAGAAGT
MMP2	GCGGCGGTCACAGCTACTT	TTCAGACTTTGGTTCTCCAGCTT
MMP9	GGACGATGCCTGCAACGT	GTACTTCCCATCCTTGAACAAATACA
MT1MMP	GAAGGATGGCAAATTCGTCTTC	AGGGACGCCTCATCAAACAC
β1-integrin	TTGGATTCTCCAGAAGGTGGTT	TCAGTGATCCACAAACTGCAACT
α1-integrin	TGCTCTCAATCAGACAAGGTTTG	GAGATGAACAGCACGTCTGCTT
α2-integrin	TGCCCCGAGCACATCAT	CGCAAATCCAAAGAGTTGACAA
α6-integrin	ACAGAAAGTGTGCATGGAGGAAA	TGGGAATGGGACGCAGTT
E-CADHERIN	CCAGTGAACAACGATGGCATT	TGCTGCTTGGCCTCAAAAT
N-CADHERIN	TGGGAATCCGACGAATGG	GCAGATCGGACCGGATACTG
SNAIL	CCCCAATCGGAAGCCTAACT	GCTGGAAGGTAAACTCTGGATTAGA
SLUG	TGCGGCAAGGCGTTTT	TCTCCCCCGTGTGAGTTCTAA
VIMENTIN	TCCAAACTTTTCCTCCCTGAAC	GGGTATCAACCAGAGGGAGTGA
β-ACTIN	GGTCATCACCATTGGCAATGAG	TACAGGTCTTTGCGGATGTCC

### Anticancer-drug-resistance assays

Cells (3.0 × 10^3^ cells/well) in growth medium were plated in 96-well culture plates, and 1 day later, anticancer drugs were added at 10 or 100 μM; at 4 days after plating, cell-growth rates were measured by means of ATP assays performed using a CellTiter-Glo^®^ 2.0 Assay kit (Promega, Madison, WI, USA). Cell viability was calculated as the percentage of luminescence values in drug-treated cells relative to that in non-treated control cells.

### Sphere-formation assays

To generate spheres, cells (1.0 × 10^3^ cells/well) in serum-free medium containing epidermal growth factor (20 ng/mL, R&D Systems, Inc. Westerville, OH, USA) and basic fibroblast growth factor (10 ng/mL, ReproCELL Inc. Kanagawa, Japan) were plated in 24-well ultra-low attachment plates (Corning Inc., Kennebunk, ME, USA). After 7 days, the spheres that formed were counted by using a phase-contrast microscope (Eclipse TS-100, Nikon, Tokyo, Japan).

### Invasion assays

Cell-culture inserts (pore size: 8 μm; diameter: 6 mm) coated with Matrigel were used according to manufacturer instructions. Cells were plated at a density of 1 × 10^5^ cells/500 μL on the upper surface of the inserts, and 18 h later, the cells that had migrated through the membrane to the lower surface of the filter were fixed and stained with a Diff-Quick staining kit (Polysciences, Inc., Warrington, PA, USA) and then counted under a light microscope.

### 3D invasion assays

Cells (1.0 × 10^3^ cells/well) in growth medium were plated in 96-well ultra-low attachment plates. After incubation for 7 days, the growth medium was gently removed from the spheroid-containing plates and 100 μL of Matrigel solution in PBS containing 10 ng/mL TGF-β1 was gently dispensed into the U-bottom wells. The plates were incubated at 37°C for the Matrigel solution to solidify, and then 100 μL of growth medium was gently added to each well. The spheres were photographed using a phase-contrast microscope (Nikon) at appropriate incubation times.

### MMP gelatin zymography

Medium collected from cells cultured in serum-free medium for 24 h at 37°C was concentrated using Amicon^®^ Ultra Centrifugal Filters (Merck Millipore), and protein assays were performed. The same amounts of samples were resolved on 8% SDS-PAGE gels containing 4.0 mg/mL gelatin. The gels were rinsed with wash buffer (0.05 M Tris-HCl, pH 7.5, 5 mM CaCl_2_, 1 μM ZnCl_2_, 2.5% Triton X-100) and soaked in incubation buffer (0.05 M Tris-HCl, pH 7.5, 5 mM CaCl_2_, 1 μM ZnCl_2_, 1% Triton X-100) at 37°C for 24 h. After incubation, the gels were fixed and stained with Coomassie R-250, washed, and scanned.

### Adhesion assays

For cell-adhesion assays, 96-well plates were coated with Matrigel (0.625 mg/mm^2^, Becton Dickinson) or 0.5% BSA in PBS for 30 min at 37°C, and then incubated with adhesion medium (0.5% BSA in serum-free RPMI 1640 medium) for 2 h at 37°C to block nonspecific binding. Cells were detached using Accutase^®^ cell-detachment solution and resuspended in adhesion medium, then 5 × 10^4^ cells/well were added to plates, and they were incubated for 1 h. To remove non-adherent cells, the plates were washed twice with PBS. Bound cell numbers were determined through ATP assays performed using a CellTiter-Glo^®^ 2.0 Assay kit. The percentage of adhesive cells was calculated as the percentage of luminescence values in adherent cells relative to that in total cells.

### Cell-cell adhesion assays

Cells were detached using Accutase^®^ cell-detachment solution and stained with Calcein-AM (PromoCell GmbH, Heidelberg, Germany). Following this, 5 × 10^4^ stain cells/well were added to 96-well plates, in which cells were grown to confluence, and incubated for 1 h. To remove non-adherent cells, the plates were washed twice with PBS. Next, bound cells were lysed with lysis buffer (Cell Biolabs, Inc., San Diego, CA, USA) and calcein fluorescence was measured.

### Statistical analysis

Quantitative data are presented as means ± standard deviation (SD). Differences between two groups were analyzed using the two-tailed Student's *t* test. Differences were considered significant at *P* < 0.05. Computations were performed using Microsoft Excel 2010 (Microsoft Corporation, Redmond, WA, USA).

## Abbreviations

BSA: bovine serum albumin
CSC: cancer stem cell
EMT: epithelial to mesenchymal transition
FACS: fluorescence-activated cell sorting
FITC: fluorescein isothiocyanate
lncRNAs: long ncRNAs
MFIs: mean fluorescence intensities
MMP: matrix metalloproteinase
ncRNAs: non-coding RNAs
PBS: phosphate-buffered saline
PDAC: pancreatic ductal adenocarcinoma
qRT-PCR: quantitative reverse transcription-polymerase chain reaction
Sc: scrambled sequence transfected shRNA
SD: standard deviation
sh: short hairpin
TGF-β1: transforming growth factor-β1
